# Supplementary figures and images for: Frequency of multiple changes to prespecified primary outcomes of clinical trials completed between 2009 and 2017 in German university medical centers: A meta-research study
Source: PLoS Med. 2023 Oct 31;20(10):e1004306. doi: 10.1371/journal.pmed.1004306 (PMC10645365; doi:10.1371/journal.pmed.1004306)

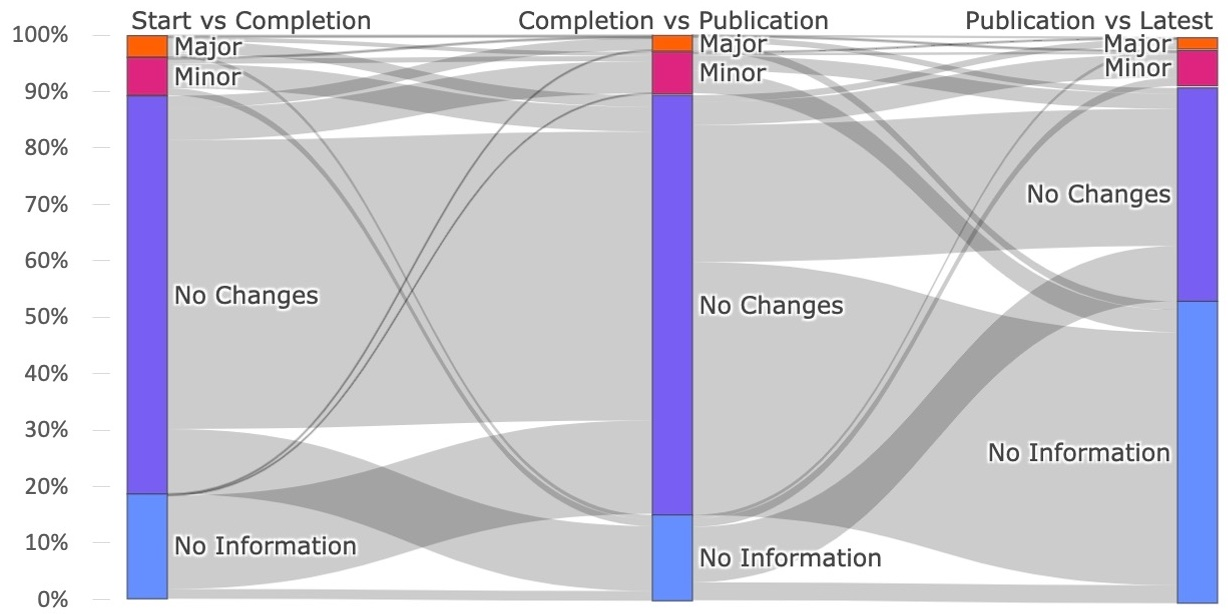

Supplement: S1 Fig — The nodes on the left show changes between the registry entry version at the start date and the registry entry version at the completion date. The nodes in the middle show changes between the registry entry at the completion date and the registry entry at the publication date. The nodes on the right show changes between the registry entry at the publication date and the latest registry entry. ‘No information’ means that the registry entries at the two timepoints were the same. This applies to circa 54% of comparisons in the right node, as the registry entry at publication is often the latest entry. The grey bars show the ‘flow’ of changes: For example, the largest grey bar between the column on the left and the column in the middle indicates that the majority of trials with no changes between the registry entry versions at the start date and completion date also have no changes between the registry entry version at completion date and publication date. For more detailed information, please see our interactive online plot: https://martin-r-h.shinyapps.io/invisibleoutcomechangesfigure/. (TIF) [file pmed.1004306.s003.tif]

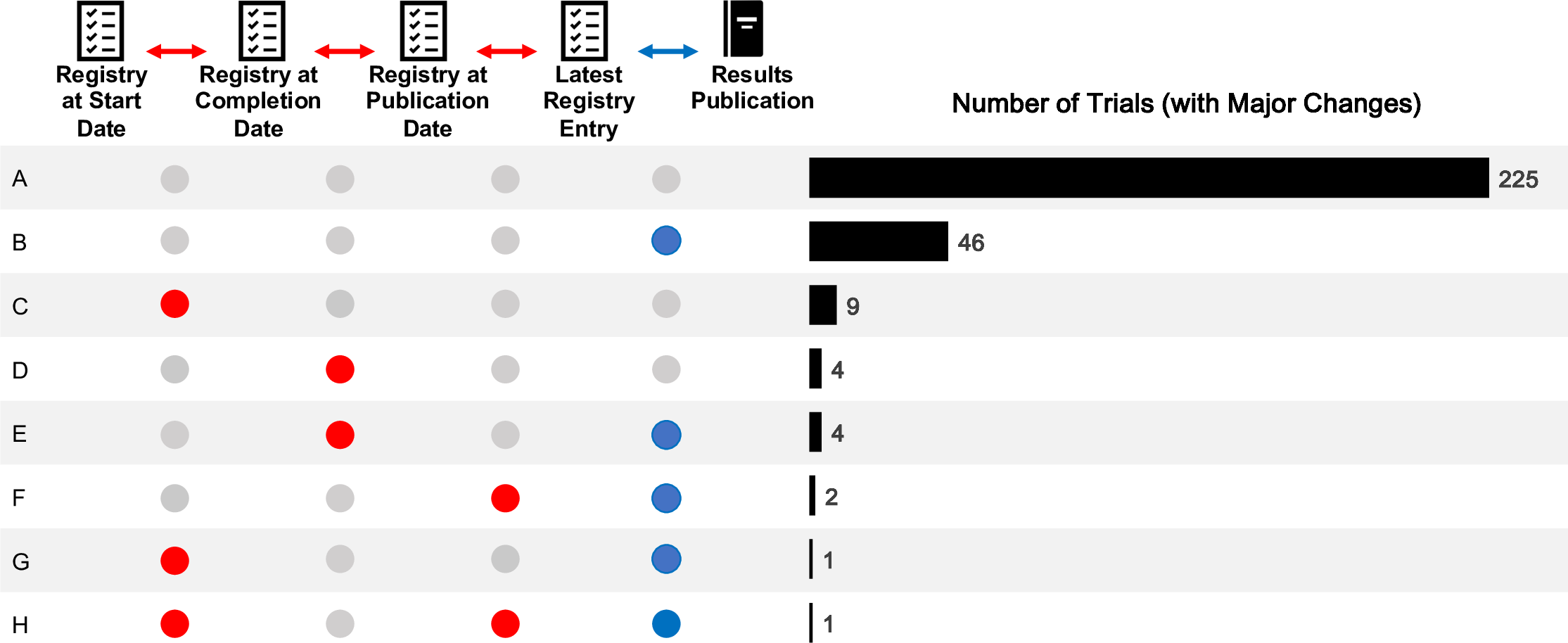

Supplement: S2 Fig — Registry entry versions were assessed at four subsequent key trial timepoints (within-registry changes; red arrows) and between the latest registry entry and results publication (blue arrow). Rows A to H show each observed combination of outcome change at the different timepoints across the 292 trials. For example, Row A indicates that 225 of 292 trials had no major outcome change (77%). Row B indicates that 46 of 292 trials only had a major change between the latest registry entry version and the results publication (blue circle; 16%). Changes in rows C and D only occurred within registry entries (red arrow, red circle) while there was agreement of the latest registry entry and the publication (no blue circle), i.e., these changes would not be detected by comparing the publication to the latest available registry entry (13 of 292 trials, 4%). Rows E, F, G and H indicate trials that had a major change between the latest entry and the published paper, but also a change at earlier timepoints. (TIF) [file pmed.1004306.s004.tif]

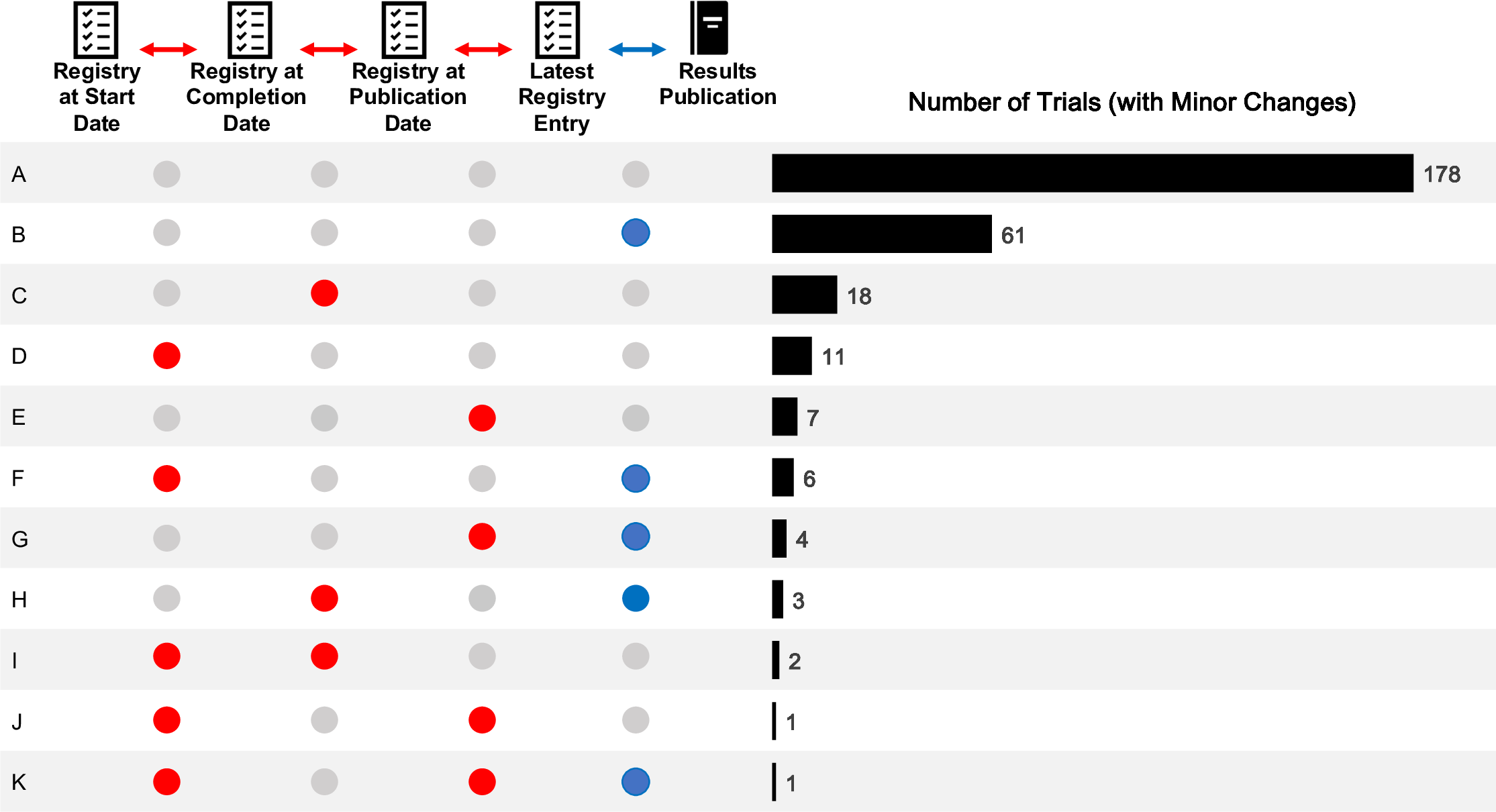

Supplement: S3 Fig — Registry entry versions were assessed at four subsequent key trial timepoints (within-registry changes; red arrows) and between the latest registry entry and results publication (blue arrow). Rows A to K show each observed combination of minor outcome change at the different timepoints across the 292 trials. For example, Row A indicates that 178 of 292 trials had no minor outcome change (61%). Row B indicates that 61 of 292 trials only had a minor change between the latest registry entry version and the results publication (blue circle; 21%). Changes in rows C, D, E, I and J only occurred within registry entries (red arrow, red circle) while there was agreement of the latest registry entry and the publication (no blue circle), i.e., these changes would not be detected by comparing the publication to the latest available registry entry (39 of 292 trials, 13%). Rows F, G, H and K indicate trials that had a minor change between the latest entry and the published paper, but also a change at earlier timepoints. (TIF) [file pmed.1004306.s005.tif]
