# Supplementary material for: Frequency of multiple changes to prespecified primary outcomes of clinical trials completed between 2009 and 2017 in German university medical centers: A meta-research study
Source: PLoS Med. 2023 Oct 31;20(10):e1004306. doi: 10.1371/journal.pmed.1004306 (PMC10645365; doi:10.1371/journal.pmed.1004306)
Supplement: S1 Table — None of the input variables had missing data, except medical field, where 74 trials could not properly be assigned. (DOCX) [file pmed.1004306.s006.docx]

Supplementary Table S1

*Holst, Haslberger, Yerunkar, Strech, Hemkens & Carlisle. Registry history changes to prespecified primary outcomes of clinical trials completed between 2009 and 2017 in German university medical centers: A meta-research study*

**S1 Table. Frequencies, odds ratios (exponentiated regression coefficients) and accompanying p-values for the logistic regression model, with any within-registry outcome change as the output variable (N = 1746 trials). None of the input variables had missing data, except medical field, where 74 trials could not properly be assigned.**

| **Input variable** | **Input variable level** | **Number of trials with any within-registry outcome change**  **(%)** | **Number of trials with no within-registry outcome change**  **(%)** | **Odds ratio**  **[95% CI]** | **p-value^1^** |
| --- | --- | --- | --- | --- | --- |
| **Study Phase** | No phase | 113 (13%) | 749 (87%) |  |  |
|  | Phase: 1 | 6 (11%) | 50 (89%) | 0.68 [0.24, 1.66] | 0.432 |
|  | Phase: 2 | 75 (28%) | 193 (72%) | 0.72 [0.44, 1.16] | 0.180 |
|  | Phase: 3 | 155 (41%) | 227 (59%) | 1.05 [0.67, 1.64] | 0.826 |
|  | Phase: 4 | 44 (25%) | 134 (75%) | 0.80 [0.49, 1.28] | 0.351 |
| **Sponsor** | Industry | 212 (47%) | 243 (53%) |  |  |
|  | Other | 181 (14%) | 1110 (86%) | 0.30 [0.21, 0.41] | < 0.001 * |
| **Publication Year (M / SD)** | --- | 2014.0 (2.6) | 2015.3 (2.5) | 1.06 [0.99, 1.13] | 0.088 |
| **Registration Year (M / SD)** | --- | 2009.3 (2.9) | 2011.9 (2.8) | 0.75 [0.70, 0.80] | < 0.001 * |
| **Medical Field** | Basic | 1 (10%) | 9 (90%) |  |  |
|  | Dentistry | 5 (20%) | 20 (80%) | 1.90 [0.22, 41.81] | 0.599 |
|  | Epidemiology and Public Health | 4 (31%) | 9 (69%) | 3.04 [0.31, 70.57] | 0.383 |
|  | Family & Reproductive Medicine | 9 (18%) | 41 (82%) | 0.82 [0.11, 16.86] | 0.864 |
|  | General Medicine | 137 (23%) | 461 (77%) | 0.92 [0.15, 17.57] | 0.935 |
|  | Health Professions | 0 (0%) | 16 (100%) | 0.00 [0.00, 0.00] | 0.969 |
|  | Immunology and Microbiology | 4 (27%) | 11 (73%) | 0.82 [0.09, 18.94] | 0.876 |
|  | Internal Medicine | 94 (28%) | 239 (72%) | 0.98 [0.16, 18.89] | 0.986 |
|  | Neuroscience | 25 (21%) | 96 (79%) | 0.96 [0.15, 18.77] | 0.968 |
|  | Nursing | 2 (10%) | 17 (90%) | 1.09 [0.09, 26.47] | 0.948 |
|  | Oncology | 41 (42%) | 56 (58%) | 1.94 [0.31, 38.06] | 0.553 |
|  | Other Clinical Field | 14 (34%) | 27 (66%) | 0.99 [0.14, 20.22] | 0.995 |
|  | Other Medical Field | 2 (6%) | 31 (94%) | 0.81 [0.06, 19.56] | 0.875 |
|  | Pharmacology, Toxicology and Pharmaceutics | 17 (15%) | 98 (85%) | 0.98 [0.15, 19.41] | 0.988 |
|  | Psychology and Psychiatry | 9 (11%) | 74 (89%) | 0.85 [0.12, 17.15] | 0.883 |
|  | Surgery | 20 (19%) | 83 (81%) | 0.80 [0.12, 15.86] | 0.844 |
|  | Other | 9 (12%) | 65 (88%) | 0.65 [0.09, 13.33] | 0.712 |
| **Registry** | ClinicalTrials.gov | 375 (27%) | 1027 (73%) |  |  |
|  | DRKS | 18 (5%) | 326 (95%) | 0.41 [0.23, 0.70] | 0.002 * |
| **Multicenter Trial** | No | 120 (13%) | 810 (87%) |  |  |
|  | Yes | 273 (33%) | 542 (67%) | 1.23 [0.90, 1.69] | 0.190 |
| **Enrollment (M / SD)** | --- | 387.6 (613.2) | 280.2 (1126.1) | 1.00 [1.00, 1.00] | 0.325 |
| **Intervention** | Device | 81 (29%) | 200 (71%) |  |  |
|  | Drug or Biological | 221 (35%) | 410 (65%) | 0.83 [0.54, 1.28] | 0.400 |
|  | Other | 91 (11%) | 743 (89%) | 0.78 [0.51, 1.20] | 0.263 |

CI: confidence-interval.

1: Asterisks indicate p-values below the common threshold for statistical significance, that is, p < 0.05.
