# Supplementary material for: Frequency of multiple changes to prespecified primary outcomes of clinical trials completed between 2009 and 2017 in German university medical centers: A meta-research study
Source: PLoS Med. 2023 Oct 31;20(10):e1004306. doi: 10.1371/journal.pmed.1004306 (PMC10645365; doi:10.1371/journal.pmed.1004306)
Supplement: S2 Table — None of the input variables had missing data, except medical field, where 9 trials could not properly be assigned. (DOCX) [file pmed.1004306.s007.docx]

Supplementary Table S2

*Holst, Haslberger, Yerunkar, Strech, Hemkens & Carlisle. Registry history changes to prespecified primary outcomes of clinical trials completed between 2009 and 2017 in German university medical centers: A meta-research study*

**S2 Table. Frequencies, odds ratios (exponentiated regression coefficients) and accompanying p-values for the logistic regression model, with any registry-publication outcome change as the output variable (n = 292 trials). None of the input variables had missing data, except medical field, where 9 trials could not properly be assigned.**

| **Input variable** | **Input variable level** | **Number of trials with any registry-publication outcome change**  **(%)** | **Number of trials with no registry-publication outcome change**  **(%)** | **Odds ratio**  **[95% CI]** | **p-value** |
| --- | --- | --- | --- | --- | --- |
| **Study Phase** | No phase | 72 (47%) | 82 (53%) |  |  |
|  | Phase: 1 | 0 (0%) | 6 (100%) | 0.00 [NA, 409458462622498662246599075268328904560937693923600498688.00] | 0.990 |
|  | Phase: 2 | 16 (42%) | 22 (58%) | 1.11 [0.39, 3.16] | 0.840 |
|  | Phase: 3 | 20 (32%) | 42 (68%) | 0.58 [0.27, 1.43] | 0.240 |
|  | Phase: 4 | 12 (38%) | 20 (62%) | 0.75 [0.29, 1.89] | 0.551 |
| **Sponsor** | Industry | 27 (39%) | 42 (61%) |  |  |
|  | Other | 93 (42%) | 130 (58%) | 0.77 [0.36, 1.66] | 0.507 |
| **Publication Year (M / SD)** | --- | 2015.0 (2.9) | 2015.3 (2.5) | 0.96 [0.84, 1.10] | 0.562 |
| **Registration Year (M / SD)** | --- | 2011.3 (3.0) | 2011.5 (2.9) | 0.92 [0.80, 1.06] | 0.278 |
| **Medical Field** | Basic | 0 (0%) | 2 (100%) |  |  |
|  | Dentistry | 2 (67%) | 1 (33%) | 16970962.28 [0.00, NA] | 0.994 |
|  | Epidemiology and Public Health | 4 (100%) | 0 (0%) | 504604443759432.00 [7093029051137710068077898569043995863185952624531832300224781878284989232313000293040128.00, NA] | 0.991 |
|  | Family & Reproductive Medicine | 6 (50%) | 6 (50%) | 13588548.38 [0.00, NA] | 0.994 |
|  | General Medicine | 36 (39%) | 57 (61%) | 7402830.67 [0.00, NA] | 0.994 |
|  | Health Professions | 1 (25%) | 3 (75%) | 2509185.26 [0.00, NA] | 0.995 |
|  | Immunology and Microbiology | 1 (100%) | 0 (0%) | 381965688152304.69 [0.00, NA] | 0.994 |
|  | Internal Medicine | 22 (34%) | 43 (66%) | 6041241.79 [0.00, NA] | 0.994 |
|  | Neuroscience | 9 (47%) | 10 (53%) | 8806614.91 [0.00, NA] | 0.994 |
|  | Nursing | 0 (0%) | 1 (100%) | 0.26 [0.00, NA] | 1.000 |
|  | Oncology | 6 (38%) | 10 (62%) | 9239658.45 [0.00, NA] | 0.994 |
|  | Other Clinical Field | 2 (67%) | 1 (33%) | 21710002.94 [0.00, NA] | 0.994 |
|  | Other Medical Field | 1 (25%) | 3 (75%) | 4808116.87 [0.00, NA] | 0.995 |
|  | Pharmacology, Toxicology and Pharmaceutics | 4 (20%) | 16 (80%) | 2922961.68 [0.00, NA] | 0.995 |
|  | Psychology and Psychiatry | 7 (54%) | 6 (46%) | 13663902.94 [0.00, NA] | 0.994 |
|  | Surgery | 14 (61%) | 9 (39%) | 13134400.18 [0.00, NA] | 0.995 |
|  | Other | 5 (56%) | 4 (44%) | 20262136.44 [0.00, NA] | 0.994 |
| **Registry** | ClinicalTrials.gov | 92 (38%) | 148 (62%) |  |  |
|  | DRKS | 28 (54%) | 24 (46%) | 1.88 [0.88, 4.05] | 0.104 |
| **Multicenter Trial** | No | 74 (48%) | 81 (52%) |  |  |
|  | Yes | 46 (34%) | 91 (66%) | 0.56 [0.29, 1.08] | 0.084 |
| **Enrollment (M / SD)** | --- | 202.4 (308.9) | 298.8 (580.1) | 1.00 [1.00, 1.00] | 0.256 |
| **Intervention** | Device | 21 (45%) | 26 (55%) |  |  |
|  | Drug or Biological | 32 (33%) | 64 (67%) | 0.65 [0.26, 1.62] | 0.357 |
|  | Other | 67 (45%) | 82 (55%) | 0.75 [0.34, 1.66] | 0.474 |

CI: confidence-interval.
