# Supplementary material for: Frequency of multiple changes to prespecified primary outcomes of clinical trials completed between 2009 and 2017 in German university medical centers: A meta-research study
Source: PLoS Med. 2023 Oct 31;20(10):e1004306. doi: 10.1371/journal.pmed.1004306 (PMC10645365; doi:10.1371/journal.pmed.1004306)
Supplement: S3 Table — The input variable did not have missing data. (DOCX) [file pmed.1004306.s008.docx]

Supplementary Table S3

*Holst, Haslberger, Yerunkar, Strech, Hemkens & Carlisle. Registry history changes to prespecified primary outcomes of clinical trials completed between 2009 and 2017 in German university medical centers: A meta-research study*

**S3 Table. Frequencies, odds ratios (exponentiated regression coefficients) and accompanying p-values for the logistic regression model, with any within-registry outcome change as the output variable (n = 292 trials) and any within-registry outcome change as input variable. The input variable did not have missing data.**

| **Input variable** | **Input variable level** | **Number of trials with any registry-publication outcome change**  **(%)** | **Number of trials with no registry-publication outcome change**  **(%)** | **Odds ratio**  **[95% CI]** | **p-value** |
| --- | --- | --- | --- | --- | --- |
| **Any Within-Registry Change** | No change | 95 (42%) | 131 (58%) |  |  |
|  | Any change | 25 (38%) | 41 (62%) | 0.84 [0.47, 1.47] | 0.546 |

CI: confidence-interval.
