# Supplementary material for: Frequency of multiple changes to prespecified primary outcomes of clinical trials completed between 2009 and 2017 in German university medical centers: A meta-research study
Source: PLoS Med. 2023 Oct 31;20(10):e1004306. doi: 10.1371/journal.pmed.1004306 (PMC10645365; doi:10.1371/journal.pmed.1004306)
Supplement: S4 Table — This table includes a sensitivity analysis for the registry-publication changes, including only publications with explicitly named primary outcomes (n = 243). (DOCX) [file pmed.1004306.s009.docx]

Supplementary Table S4

*Holst, Haslberger, Yerunkar, Strech, Hemkens & Carlisle. Registry history changes to prespecified primary outcomes of clinical trials completed between 2009 and 2017 in German university medical centers: A meta-research study*

**S4 Table. Primary outcome changes in 1746 randomized controlled trials completed between 2009 and 2017 in German university medical centers. This table includes a sensitivity analysis for the registry-publication changes, including only publications with explicitly named primary outcomes (n = 243).**

|  | **Changes within registry entries (n=1746)**  **Number of trials (%)** | | | |  | **Registry-publication changes^7^**  **Number of trials (%)**  **[95% CI]**  **(n=292)** | **Registry-publication changes^7^**  **Number of trials (%)**  **[95% CI]**  **(n=243; sample with only explicitly named outcomes)** |
| --- | --- | --- | --- | --- | --- | --- | --- |
|  | **Any^2^** | **Start date^3^ vs Completion date^4^** | **Completion date^4^ vs Publication date^5^** | **Publication date^5^ vs Latest entry^6^** |  |  |  |
| **Changes^1^** |  |  |  |  |  |  |  |
| **any** | **393 (23%)** | **167 (10%)** | **159 (9%)** | **131 (8%)** |  | **120 (41%)**  **[35%, 47%]** | **86 (35%)**  **[29%, 42%]** |
| major | 142 (8%) | 66 (4%) | 49 (3%) | 36 (2%) |  | 54 (18%)  [14%, 23%] | 32 (13%)  [9%, 18%] |
| - addition of primary outcomes | 121 (7%) | 54 (3%) | 42 (2%) | 31 (2%) |  | 36 (12%)  [9%, 17%] | 18 (7%)  [4%, 11%] |
| - deletion of primary outcomes | 57 (3%) | 25 (1%) | 19 (1%) | 14 (1%) |  | 34 (12%)  [8%, 16%] | 20 (8%)  [5%, 12%] |
| minor | 318 (18%) | 117 (7%) | 130 (7%) | 110 (6%) |  | 75 (26%)  [21%, 31%] | 59 (24%)  [19%, 30%] |
| - changes to relevant details | 149 (9%) | 49 (3%) | 61 (3%) | 51 (3%) |  | 45 (15%)  [11%, 20%] | 34 (14%)  [10%, 19%] |
| - addition/omission of relevant details | 233 (13%) | 78 (4%) | 91 (5%) | 80 (5%) |  | 32 (11%)  [8%, 15%] | 27 (11%)  [7%, 16%] |
| **No information in registry^8^** | **5 (0%)** | **332 (19%)** | **266 (15%)** | **945 (54%)** |  | **0 (0%)**  **[0%, 0%]** | **0 (0 %)**  **[0%, 0%]** |
| **none** | **1348 (77%)** | **1247 (71%)** | **1321 (76%)** | **670 (38%)** |  | **172 (59%)**  **[53%, 65%]** | **157 (65%)**  **[58%, 71%]** |

CI: confidence-interval.

1: Multiple changes may occur at different timepoints. A trial can have both major and minor changes. Categories in boldface are mutually exclusive and add up to 1746 or 292, respectively.

2: Any changes to primary outcomes reported in the registries at different trial timepoints (within-registry changes)

3: Start date (i.e., the registry entry version at the time of first patient inclusion).

4: Completion date (i.e., the last registry entry version before the primary completion date).

5: Publication date (i.e., the registry entry version at the date of the first results publication).

6: Latest entry (i.e., the most recent registry entry version by the time we retrieved the data).

7: Registry-publication changes refer to differences between the latest registry entry and the published paper.

8: Milestone does not exist in registry.
