# Supplementary material for: Frequency of multiple changes to prespecified primary outcomes of clinical trials completed between 2009 and 2017 in German university medical centers: A meta-research study
Source: PLoS Med. 2023 Oct 31;20(10):e1004306. doi: 10.1371/journal.pmed.1004306 (PMC10645365; doi:10.1371/journal.pmed.1004306)
Supplement: Attachment — Submitted filename: ASCERTAIN_Response_to_Reviewers_06_final.pdf [file pmed.1004306.s010.pdf]

"Frequency of 'invisible' changes to prespecified primary outcomes of clinical trials completed between 2009 and 2017 in German University Medical Centres: A meta-research study"

Holst, Haslberger, et al.

PMEDICINE-D-23-00549R2

Point-by-point reply

31 August 2023

NR	AREA	COMMENT (Editor)	RESPONSE
1	GENERAL	Please justify the inclusion of studies published only from Germany.	Thank you! We now explain why we used a sample from Germany in the Introduction and the Methods (Data sources and sample):<br><br>*"We are aware of only two studies that examined changes to primary outcomes over the course of the entire registry version history. Both used relatively small and selective samples (either trials published in ICMJE journals or trials registered on ClinicalTrials.gov covered by the Food and Drug Administration Amendments Act) over a four-year time period [30,31]. We are not aware of studies quantifying the frequency of changes that may be 'invisible' and potentially unnoticed.*<br>*In this study, we aimed to provide an in-depth analysis of changes to primary outcomes using a comprehensive nationwide sample over 9 years that represents the current practice of trial registration in academic research."* (Introduction)<br><br>*"We based our analyses on previous work of our group that provided the highly granular and large-scale data on both registry entries and accompanying results publications required to address our aims. Two large*

			datasets [32,33] of randomized controlled trials covering a period of 9 years of trial completion plus a period of 5 years of publication tracking were used for this project. The trials were completed at German university medical centers between 2009 and 2017 and had been registered in either ClinicalTrials.gov or the German World Health Organization primary registry (German Clinical Trials Register or Deutsches Register Klinischer Studien; DRKS). Both registries have an accessible history of changes. The corresponding results publications were identified in manual searches up to 2022, ensuring an at least five-year period after trial completion and complementing the often inconsistent references in the trial registry [34].” (Methods)
2	GENERAL	As you report an analysis of existing studies as metadata (also noting the use of ‘Numbat Systematic Review Manager’ for data extraction), we suggest that you consider reporting your study according to PRISMA for SRMAs instead of STROBE. The PRISMA guidelines can be found at the EQUATOR site, http://www.equator-network.org/reporting-guidelines/prisma/. We acknowledge that some aspects of PRISMA reporting may not apply here.<br>Please provide the completed PRISMA checklist.<br>When completing the checklist, please use section and paragraph numbers, rather than page/line numbers as these often change in the event of publication.<br>Please add the following statement, or similar, to the Methods: "This study is reported as per the Preferred Reporting Items for Systematic Reviews and Meta-Analyses (PRISMA) guideline (S1 Checklist)."	We have carefully perused PRISMA and ensured that all applicable items are adequately reported in the revised version of the manuscript; we are submitting these files along with our revisions. We would prefer to also use the STROBE guideline as we feel it also adds important details. We employed the STROBE guideline in many other similar types of cross-sectional meta-research studies, also for a recent publication from our team in PLOS Medicine:<br>Franzen DL, Carlisle BG, Salholz-Hillel M, Riedel N, Strech D (2023) Institutional dashboards on clinical trial transparency for University Medical Centers: A case study. PLOS Medicine 20(3): e1004175. https://doi.org/10.1371/journal.pmed.1004175
3	GENERAL	Please temper the language used throughout, including the use of the word ‘hidden’ in the title, and ensure only objective reporting of your findings and their implications.	Thank you for highlighting this important point! It was not our intention at all to suggest ‘malfeasance’ and we have changed the word in the title to ‘invisible’.<br>Please note, that by using the term ‘hidden’, we did not mean to imply that researchers purposely hide important information in registry histories, but rather that the information is hidden.

4	GENERAL	Comments from the Academic Editor - it would be important for the authors to avoid words like "Hidden" in the title, which implies malfeasance when we don't know either way about that. A chance for major revision seems reasonable to me.	We have carefully perused the text and adapted it accordingly to avoid any impression of malfeasance. Please also see response to #3 above.
5	DECLARATIONS	Lines 44-60, please remove these statements from the main manuscript and include only in the manuscript submission form. They will be compiled as metadata in the event of publication.	We changed it accordingly.
6	TITLE	Please remove the word 'hidden' from your title and revise it according to PLOS Medicine's style. Your title must be nondeclarative and not a question. It should begin with main concept if possible. "Effect of" should be used only if causality can be inferred, i.e., for an RCT. Please place the study design ("A randomized controlled trial," "A retrospective study," "A modelling study," etc.) in the subtitle (ie, after a colon).	We now changed the title accordingly: *"Frequency of 'invisible' changes to prespecified primary outcomes of clinical trials completed between 2009 and 2017 in German university medical centers: A meta-research study"*
7	ABSTRACT	Please report your abstract according to PRISMA for abstracts, following the PLOS Medicine abstract structure (Background, Methods and Findings, Conclusions). Further details can be found via this link http://www.plosmedicine.org/article/info:doi/10.1371/journal.pmed.1001419	We now modified the abstract accordingly.
8	ABSTRACT	Abstract Background:<br>Please provide the context of why the study is important. The final sentence should clearly state the study question	We now modified the abstract and state:<br>*"Clinical trial registries allow to assess deviations of published trials from their protocol, which may indicate a serious risk of bias. However, since entries in many registries can be updated at any time, deviations may go unnoticed. We aimed to assess the frequency of changes to primary outcomes in different historical versions of registry entries, and how often they would go unnoticed if only deviations between published trial reports and the most recent registry entry are assessed."*
9	ABSTRACT	Abstract Methods and Findings:<br>Line 68 – please amend to read 'randomized controlled trials'	We changed it to "randomized controlled trial" throughout the manuscript accordingly. We also added the following to the Abstract's eligibility criteria:<br>*"We analyzed the complete history of changes of registry entries in all 1746 randomized controlled trials completed*

			at German university medical centers between 2009 and 2017…"
10	ABSTRACT	Please ensure that all numbers presented in the abstract are present and identical to numbers presented in the main manuscript text.	Thank you! We carefully checked our abstract and ensured that the numbers and reporting is consistent.
11	ABSTRACT	Please provide the eligibility criteria, and synthesis/appraisal methods.	We adapted the abstract and now state:<br>*"We analyzed the complete history of changes of registry entries in all 1746 randomized controlled trials completed at German university medical centers between 2009 and 2017, with published results up to 2022, that were registered in ClinicalTrials.gov or the German WHO primary registry (German Clinical Trials Register; DRKS). Data were retrieved on 24 January 2022. We assessed deviations between registry entries and publications in a random subsample of 292 trials. We determined changes of primary outcomes (1) between different versions of registry entries at key trial milestones, (2) between the latest registry entry version and the results publication, and (3) 'invisible' changes (changes after trial start with no change between last registry entry version and publication, so that assessing the full history of changes is required for detection of changes). We categorized changes as major if primary outcomes were added, dropped, changed to secondary outcomes, or secondary outcomes were turned into primary outcomes. We also assessed (4) the proportion of publications transparently reporting changes and (5) characteristics associated with changes."*
12	ABSTRACT	Please justify the choice of start and end dates of the studies included/searched for.	We adapted the abstract, see #11. In the methods section of the main text, we now describe in more detail the selection criteria (we aimed for a long time period of almost a decade providing a large sample and we ensured a sufficient period post-completion of 5 years to identify any available results publication).
13	ABSTRACT	Please quantify the main results p values as well as with 95% CIs.	We carefully checked our Abstract and now report p-values for our regression analyses and 95% CIs for our

			subsample of 292 publications, to quantify possible sampling error.<br>As our main dataset of 1746 trials constitutes a full population, we believe that confidence intervals would not be required. If this issue is considered important by the Editors or Reviewers, of course, we can adapt this if deemed necessary.
14	ABSTRACT	When reporting p values please report as p<0.001 and where higher as p=0.002, for example.	We changed this accordingly.
15	ABSTRACT	Suggest reporting statistical information as follows – 'OR 0.29; 95% CI [0.21, 0.41], p<0.001'	We changed this accordingly.
16	ABSTRACT	Please define OR at first use for the reader.	We now define the abbreviations accordingly.
17	ABSTRACT	Were any variable factors adjusted for in your analyses? If so, please detail which.	We performed a single exploratory logistic regression analysis. We now clearly report this:<br>"*An exploratory logistic regression analysis indicated that trials were less likely to have a discrepant registry entry if they were registered more recently (odds ratio (OR) 0.74; 95% CI [0.69, 0.80]; p<0.001), were not registered on ClinicalTrials.gov (OR 0.41; 95% CI [0.23, 0.70]; p=0.002), or were not industry-sponsored (OR 0.29; 95% CI [0.21, 0.41]; p<0.001).*"
18	ABSTRACT	In the last sentence of the Abstract Methods and Findings section, please describe the main limitation(s) of the study's methodology.	We now describe the main limitations and state:<br>"*Key limitations include some degree of subjectivity in the categorization of outcome changes and inclusion of a single geographic region.*"
19	ABSTRACT	Abstract conclusions:<br>Please temper the language used here and address the study implications without overreaching what can be concluded from the data; the phrase "In this study, we observed ..." may be useful.	Thank you very much for this suggestion; we toned down the language, starting the conclusion with "*In this study, we observed that changes to primary outcomes are common and often major.*".
20	ABSTRACT	Please interpret the study based on the results presented in the abstract, emphasizing what is new without overstating your conclusions.	We adapted the text accordingly. See response above (#19).
21	ABSTRACT	Please avoid vague statements such as "these results have major implications for policy/clinical care". Mention only specific implications substantiated by the results.	The term 'major relevance' in the abstract conclusion refers to the quality of outcome change (i.e., major outcome changes), not implications for policy/clinical

			care. We agree that this was misleading. We changed the wording to be more descriptive:<br>*“In this study, we observed that changes to primary outcomes are common and often major.”*
22	ABSTRACT	Please avoid assertions of primacy ("We report for the first time....")	We carefully perused the abstract and main text to avoid assertions of primacy. In the Introduction we mention that we are not aware of other research quantifying the frequency of potentially unnoticed changes by considering only the latest registry entry to reflect the existing evidence (please also see comment #24).
23	AUTHOR SUMMARY	At this stage, we ask that you include a short, non-technical Author Summary of your research to make findings accessible to a wide audience that includes both scientists and non-scientists. The authors summary should consist of 2-3 succinct bullet points under each of the following headings:<br>• Why Was This Study Done? Authors should reflect on what was known about the topic before the research was published and why the research was needed.<br>• What Did the Researchers Do and Find? Authors should briefly describe the study design that was used and the study’s major findings. Do include the headline numbers from the study, such as the sample size and key findings.<br>• What Do These Findings Mean? Authors should reflect on the new knowledge generated by the research and the implications for practice, research, policy, or public health. Authors should also consider how the interpretation of the study’s findings may be affected by the study limitations. In the final bullet point of ‘What Do These Findings Mean?’, please describe the main limitations of the study in non-technical language.<br>The author Summary should immediately follow the Abstract in your revised manuscript. This text is subject to editorial change and should be distinct from the scientific abstract. Please see our author guidelines for more information: https://journals.plos.org/plosmedicine/s/revising-your-manuscript#loc-author-summary	We now include a non-technical summary after the Abstract (see page 5)

24	INTRODUCTION	Please address past research and explain the need for and potential importance of your study. Indicate whether your study is novel and how you determined that. If there has been a systematic review of the evidence related to your study (or you have conducted one), please refer to and reference that review and indicate whether it supports the need for your study.	Thank you, we have indeed not reported this adequately. We have revised the penultimate paragraph of the Introduction in which we put our research in context and now explicitly state that we have done a systematic literature search. This also included some backward citation tracking. We have now uploaded our search filters and results of the structured data extractions to the project's OSF repository and cite this accordingly (https://osf.io/za9rx/). In the overview paragraph, we describe that comparisons of registry data to publications are often based on unclear or varying (historical) versions of trial registrations. The manuscript now reads:<br>*"We systematically searched studies that have assessed the frequency of outcome changes between registries and publications [7]. We identified several analyses which widely agree that this problem is very common, with a median estimated prevalence of 31% (interquartile range (IQR): 17-45%) of clinical trials affected [8]. However, most studies did not report which registry entry version they used [9–17], or reported that they only assessed changes between the publication and one single registry entry version (i.e., the latest available entry [18–26], the latest entry before trial completion [27], the first available entry [28], or the first entry during the active phase [29]). We are aware of only two studies that examined changes to primary outcomes over the course of the entire registry version history. Both used relatively small and selective samples (either trials published in ICMJE journals or trials registered on ClinicalTrials.gov covered by the Food and Drug Administration Amendments Act) over a four-year time period [30,31]. We are not aware of studies quantifying the frequency of changes that may be 'invisible' and potentially unnoticed."*
25	INTRODUCTION	Please remove the phrase 'cherry-picking'	We removed the phrase and replaced it with *'selective reporting of only statistically significant results'*.

26	INTRODUCTION	Line 116 – please refrain from using the word ‘hidden’.	We changed this. Please see our response to comment #3.
27	INTRODUCTION	Lines 130 onwards – please replace square brackets with circular (in-text reference callouts should be placed in square brackets – see below under REFERENCES)	We now changed the text and our citation style accordingly to meet the journal requirements and changed the square brackets to round brackets for numbering of, for example, objectives.
28	INTRODUCTION	Has there been a systematic review of the evidence related to your study (or if you have conducted one)? If so, please refer to and reference that review and indicate whether it supports the need for your study.	Please see our response to your previous comment (#24).
29	METHODS and RESULTS	Line 142 – please place citation in square brackets	We changed this (please also see comment #27).
30	METHODS and RESULTS	Line 143 – ‘interventional studies’ please replace with ‘randomized controlled trials’	We changed this accordingly.
31	METHODS and RESULTS	As above, please justify the inclusion of data from these specified time points	Thank you! We now better explain our approach in the Methods, see comment #1.
32	METHODS and RESULTS	As above, please justify the inclusion of only studies originating from Germany	This project required highly granular data on publications, which are available to us for Germany. We now better explain our approach (see comment #1 above), and also now explicitly discuss this geographic restriction in the Discussion:<br>*“Fourth, although we used high quality underlying data reflecting German clinical research output, we only assess a single geographic area, partly reflecting EU/German registration policies. Still, many registration policies are international, and our results point to the possibility that technical details of the registries rather determine whether outcome changes occur.”*<br>While we have no reason to believe that the registration of study outcomes is substantially different in other countries, we agree that our research needs to be replicated for other settings and now better highlight this limitation. The phenomenon of invisible outcome changes has, however, not been described before.

33	METHODS and RESULTS	Line 167, 213 – please use an alternative word for 'downloaded', 'extracted' perhaps? Please check and amend throughout.	Thank you for pointing this out. Since we use the term 'extract' already when we talk about extraction of primary outcomes from the dataset (in accordance with common language in systematic reviews and in the now applied PRISMA guidance), we exchanged 'downloaded' with 'retrieved' (as used by e.g. PRISMA).
34	METHODS and RESULTS	Line 170 onwards – please reserve the use of square brackets for in-test reference callouts	Changed accordingly (please also see #27).
35	METHODS and RESULTS	Line 213 onwards – details of the software packages would be better placed earlier at line 165 where you describe data extraction and processing.	Changed accordingly.
36	METHODS and RESULTS	Line 216 – suggest reporting according to PRISMA instead of STROBE.	We now also use PRISMA (please also see #2).
37	DISCUSSION	Please remove the sub-heading 'conclusion' form the end of the discussion.	Changed accordingly.
38	DISCUSSION	As above, please refrain from using the word 'hidden'.	Changed accordingly (please also see #3).
39	REFERENCES	For in text reference callouts please place citations in square brackets.	Changed accordingly (please also see #27).
40	REFERENCES	Please list up to but no more than 6 author names followed by et al.	Changed accordingly.
41	REFERENCES	Please ensure that all web references include an access date. Please see our website for other reference guidelineshttps://journals.plos.org/plosmedicine/s/submission-guidelines#loc-references<https://journals.plos.org/plosmedicine/s/submission-guidelines#loc-references>	Changed accordingly.
42	TABLES	Please ensure that all tables area affiliated to an appropriate caption which clearly describes the table contents without the need to refer to the text. Please ensure all abbreviations are defined for the reader.	We made these changes accordingly.
43	TABLES	Suggest presenting the trial characteristics as table 1 followed by the definitions of discrepancies as table 2.	Thank you for this suggestion! We changed the order of the tables accordingly.
44	TABLES	Table 1 – outcome discrepancies – these seem rather vaguely defined, suggest additional nuance to the categorization. When reporting minor discrepancies the word 'significant' is used implying a major discrepancy and is also not very quantified. Please revise this table including more nuanced detail of the categorization applied.	Thank you for this important comment. We fully agree that the word significant in combination with minor discrepancies is confusing! We removed it.

			We also revised the table substantially and refer to related literature using similar categorization and provide more details the Methods:<br>"*Reviewers assessed changes to the primary outcomes between the four key trial milestones within the registry (Figure 1), using a classification system that categorizes changes to primary outcome as either major changes (based on an existing classification system [40]) or minor changes (adapted from [21] and [41], for details see Table 2).*"<br>Please see also comment #94 by Reviewer 2.
45	TABLES	Table 2 – The category 'other' constitutes the second large group of studies in your dataset. Could other be sub-categorized for additional information?	Thank you very much for pointing this out! The medical field categories were determined by matching the trial's journal name with journal field information from the Scimago Country & Rank database. The 'other studies' were trials where no matching journal had been found using our automated approach. We now manually identified the respective journal matches, and thus, the number of 'others' has reduced from 395 to 74. Please note that due to these changes, we updated our regression models (no relevant changes, conclusions remain the same).
46	TABLES	Table 3 – for the main outcomes measures please indicate whether your analyses are adjusted or unadjusted and if adjusted please present the unadjusted analyses for comparison. Please report p as p<0.001 and where higher the exact p value. Please define the meaning of the lettering in bold type-face	The analyses in Table 3 do not include statistical tests or models, we have no p-values to report. We now explain the meaning of boldface lettering in the caption, thank you for bringing this up!
47	FIGURES	Please ensure that all figures are associated with a caption that clearly describes the figure content without the need to refer to the manuscript text. Please ensure all abbreviations and the meaning of dots, lines and bars are clearly defined for the reader.	We have carefully revisited all figures, legends and captions to ensure these issues are adequately addressed.
48	FIGURES	Figure 1 – if the end point in this flow diagram is 'journal publication' does the earlier 'publication' refer to the protocol publication? Please clarify/revise for the reader.	The figure has been substantially revised following the suggestions by the Editors and Reviewers.

			We clarified the text in the Figure to reflect that '*journal publication*' refers to the *registry entry version at the time of journal publication*.<br>Aiming to substantially improve clarity, we ensured that the Figures are better harmonized, and we now closely align Figure 1 with Figure 3 (the former Figure 4) with regard to colors, descriptions, and symbols used.
49	FIGURES	Figure 3 is striking but rather confusing. It details percentages but does not give a scale to determine the percentage. What do the different widths of shaded grey areas and lines represent? Perhaps a key for the colored bars could be provided as a legend for clarity.	The figure has been substantially revised following the suggestions by the Editors and Reviewers. Additionally, we have moved it to the supplement (now as Supplementary Figure 1).<br>The different widths of shaded grey areas and the lines represent the 'flow' of changes: For example, the largest grey bar between the column on the left and the column in the middle indicates that the majority of trials with no changes in the registry between study start and study completion also have no changes in the registry between completion and publication. We have explained this in the caption.<br>We agree that this figure is challenging. To improve it, we have now added a scale to show the percentages, changed the naming of the colored bars, and changed the legend. We have also created an online, interactive version: https://martin-r-h.shinyapps.io/InvisibleOutcomeChangesFigure/
50	FIGURES	Figure 4 – please provide the unit of measurement for the 'discrepancies across timepoints' total number? The label of the y axis could be closer to the graph, please revise.	Thank you for your comments! Following your feedback, as well as feedback from the reviewers, we have now completely reworked Figure 4 (now Figure 3).
51	SUPPLEMENTARY FIGURES	Please ensure that all figures are associated with a caption that clearly describes the figure content without the need to refer to the manuscript text.	We made the requested changes to our caption and legend.
52	SUPPLEMENTARY FIGURES	Please ensure all abbreviations and the meaning of dots, lines and bars are clearly defined for the reader.	We made the requested changes to our caption and legend.
53	SUPPLEMENTARY FIGURES	Please ensure axis labels are placed appropriately alongside the relevant axis.	We changed this accordingly.

54	SUPPLEMENTARY FIGURES	Please ensure that all axes have a defined unit of measurement e.g., total number.	We have changed the descriptions of the Figures accordingly.
55	SUPPLEMENTARY FIGURES	If using 'disc.' as an abbreviation please ensure that it is defined,	We completely revised the figures, see comment #50.
56	SUPPLEMENTARY TABLES (56)	Where p values are presented, please report as p<0.001 and where higher as the exact p value. Please also include 95% CIs where you report p values.	Changed accordingly, where applicable.
**NR**	**AREA**	**COMMENT (Reviewer 1)**	**RESPONSE**
	GENERAL	This paper reports an investigation of discrepancies between registration entries and publications in clinical trials in terms of primary outcomes. The authors demonstrate there are a considerable number of discrepancies and that the type of study is associated with the chance of discrepancy. The statistical methods used are (appropriately) straightforward. I had some comments on the reporting below:	We are very grateful to the reviewer for his very careful review and constructive input. Thank you!
57	Abstract	'[1] Primary outcome discrepancies between registry entries at key study milestones and [2] the first results publication." - possibly moving the [1] might help this be a bit clearer, e.g.: 'Primary outcome discrepancies between registry entries [1] at key study milestones and [2] the first results publication."	Thank you for this suggestion. We changed the text accordingly.
58	Abstract	"Only 1% of discrepancies were reported in the publications (2/161, 95% CI [0%, 4%])." - can it be made clearer from the abstract why 161 is the denominator here?	The denominator of 161 is the number of trials with any outcome change, and thus the number of trials for which we would expect some kind of reporting of changes. We now made this clearer:<br>*"Only 1% of trials with a change reported this in their publication (2 of 161 trials; 95% CI [0%, 4%])."*
59	Abstract (and results)	the ORs are <1 indicating registry discrepancy is less likely, not more likely	We agree, the odds ratios below 1 indicate that changes are less likely, and this can be confusing. We now have inverted our statement by changing "earlier" to "later" registration and adding "not" to ClinicalTrials.gov registration and Industry sponsorship; see also comment #17:<br>*"An exploratory logistic regression analysis indicated that trials were less likely to have a discrepant registry entry if*

			they were registered more recently (odds ratio (OR) 0.74; 95% CI [0.69, 0.80]; p<0.001), were not registered on ClinicalTrials.gov (OR 0.41; 95% CI [0.23, 0.70]; p=0.002), or were not industry-sponsored (OR 0.29; 95% CI [0.21, 0.41]; p<0.001).”
60	Methods (and discussion)	the agreement for registry-publication results seems fairly low - this is presumably prior to discussion to resolve disagreements? This does seem a limitation to applying these results in practice for identifying whether there were inconsistencies when reviewing future publications: some reviewers may identify an issue and some may not. Perhaps this could be mentioned in the discussion.	This is an excellent point, thank you! Yes, we report the inter-rater agreement prior to the consensus discussion. However, our detailed and fine-grained rating terminology (we differentiate 8 categories of changes) is probably not necessary for the peer review process where the larger changes may matter more.<br>We now briefly add that the subjectivity in the assessment may nevertheless translate to the peer-review process in the Discussion section:<br>*“However, conflicting primary outcome definitions and conflicting ratings were resolved in discussions between the reviewers (and, where necessary, a third reviewer), and excluding unclear situations in the above-mentioned sensitivity analysis did not change the main findings. Still, this could become an issue in practice if, for example, different peer-reviewers disagree in their assessment of whether an outcome change has happened or not.”*
61	Discussion	I presume the authors did not extract information on whether results were significant with the discrepant outcomes. This would be interesting information if it exists (e.g. trials with discrepant outcomes were more likely to report significant results) as it would imply that this might involve cherry-picking.	Thank you for this suggestion. We did extract this information for some of the sampled studies, but a thorough analysis would be beyond the scope of the current project – we would think that the significance of the original outcome would also be relevant. We plan to explore this in a subsequent project.
62	Supplementary table 2	it would be useful to provide the 95% CI for the odds ratios in this table.	We have added the confidence intervals, thank you for this suggestion! (See also comment #56 from the Editor.)
**NR**	**AREA**	**COMMENT (Reviewer 2)**	**RESPONSE**
63	GENERAL	This is an interesting meta-research study investigating discrepancies between clinical trial protocols in registries vs published reports.	Thank you for this very helpful feedback.

		Overall, the findings are likely to be of interest to trialists and other readers, though the manuscript and reporting could be improved in a number of areas. Please see comments below.	
64	GENERAL	The scope of this study is substantial, with a large number of outcomes, statistical tests, and presentation in figures that are not common in most fields of research. In addition, the writing uses a number of assumed terms and knowledge, which may be problematic for the broad readership of this journal. And the writing is hard to follow in places.	We carefully revised our manuscript and hope it is easier to follow now.
65	GENERAL	* Suggest to edit the manuscript to simplify wording and tighten the writing, remove/minimise jargon, and clearly define terms at first use.	We have carefully revised our text, aiming to make it more readable, removing jargon and providing more examples and illustrative language.
66	GENERAL	* Consider displaying data using figures that are more recognisable in medical research. If not possible, substantially expand the figure legends to orientate figures to readers who may not be research-active in this area.	We agree that these figures are uncommon. We took great thought into the visualization of the data and now adapted them to facilitate their usefulness for medical/clinical readers. We now have extensively explained the Figures and their legends (see the more detailed comments below).
67	ABSTRACT	L64. ‘primary outcome discrepancies’ can read as jargon: consider wording as e.g. ‘differences between primary outcomes reported in trial registries and in published reports (i.e. primary outcome discrepancies)’	We revised this accordingly across the manuscript and now state: *“We determined changes of primary outcomes (1) between different versions of registry entries at key trial milestones, (2) between the latest registry entry version and the results publication, and…”*.
68	ABSTRACT	L64. The first sentence also seems odd: It is worded as “to asses. discrepancies within registry records..” but does not state what registry records are discrepant to. Please refine.	We revised this sentence accordingly and now state: “*We aimed to assess the frequency of changes to primary outcomes in different historical versions of registry entries, and how often they would go unnoticed if only deviations between published trial reports and the most recent registry entry are assessed.*”
69	ABSTRACT	L77. We considered discrepancies [ADD: to be] major if primary outcomes were [DEL: newly] added, dropped, [EDITED: replaced by secondary outcomes], or [EDITED: used to replace secondary outcomes].	Thank you for this helpful suggestion. We changed the sentence as follows: *“We categorized changes as major if primary outcomes were added, dropped, changed to secondary outcomes,*

			or secondary outcomes were turned into primary outcomes."
70	ABSTRACT	L82 and in Tables, Results throughout manuscript: A 95% CI represents a range of values over which the estimate falls. Please format all confidence intervals as e.g. [35% to 47%], instead of using commas to separate the upper and lower limits. This makes clear to readers that the confidence interval is a range of values.	Please see comment #15 by the editor. While we agree that this would be more intuitive, we adhered to the suggestion by the editor and kept the version with commas.
71	INTRODUCTION	L98. The word 'critical' is mentioned 3 times in the first 6 lines, which seems an overuse of the word and dilutes its seriousness. Please rephrase to keep the emphasis on what is important and de-emphasise less important points.	We agree and rephrased the text:<br>*"Clinical trial registries are tools to enhance the transparency and trustworthiness of clinical trial evidence by openly reporting key study parameters in a rapidly accessible manner [1]. They make it possible to detect selective reporting biases that pose a threat to trustworthiness, by allowing reviewers to assess possible deviations from an original protocol and analysis plan. One of the most critical deviations is a change to prespecified outcome measures (or, 'outcomes', for short). Consequently, outcomes and their pre-specification are among the most important reporting items of clinical trials, with the CONSORT statement asking authors to…"*
72	INTRODUCTION	L103. .. asking [ADD: authors] to	We rephrased the text accordingly.
73	INTRODUCTION	L106: 'changed': what does the change represent? I.e. changed from __ to __?	We rephrased the text to be more precise: "*If readers do not know whether there have been any changes to the outcomes, e.g., by adding or dropping primary outcomes, they cannot assess the risk of bias due to selective reporting of only statistically significant results or multiple hypothesis testing [3]."*
74	INTRODUCTION	L107: What are the implications of multiple testing?<br>For that matter, on my count there are 7 + 28 + 27 + 1 + 7 = 70 tests in this study, in the main paper and supplementary tables. This large number of tests highlights the exploratory nature of this study, and also makes it prone to limitations of multiple testing.<br>Why might this study fair better than other studies with substantial multiple tests?	Thank you for raising this important point! In our view, the framework of a clinical trial that aims to determine the better treatment choice with usually a single clinical question (in the PICO framework; usually with a single primary outcome and a single primary analysis) is very different to the design of this study that explores various research questions on different issues. However, we

			definitely agree that such challenges apply to all research, including ours. We preemptively addressed them by aiming for maximal transparency in our processes. Our study was preregistered, has a published protocol, and we report deviations from the plan in our protocol amendment.<br><br>Please note, however, that we highlight the problems resulting from **undisclosed** multiple testing, and selective reporting. We also added this aspect to the Discussion, as well as more background information on the implications of outcome switching and analytical choices, which are related to multiple testing:<br><br>*"To conclude, primary outcome changes are very common in clinical research. Such changes are often, in one of four cases, 'invisible' in the trial registries and almost never reported in publications. While changes to primary outcomes are not unusual in clinical trials and may be well justified and sometimes even desirable, undisclosed changes are a problem."*
75	INTRODUCTION	L115: What is being considered?	We added 'trial registries' to make this clearer.
76	INTRODUCTION	L122-124. The flow between sections of this sentence is not clear 'most studies published to date did not XXX, assessed XXX, or the latest entry ...' does not make sense. Please fix.	We adapted the flow of sentences in that section.
77	INTRODUCTION	L126-129: It seems unclear what value these statements add to this paragraph, that there is variability between registered protocols and trial reports. Consider dropping them, or revise to connect the ideas more strongly to the sentences above.	We reworded this paragraph and deleted a sentence.
78	INTRODUCTION	L130-137. The large number of aims and lengthy articulation seems to dilute the strength of the key message of this study, and make the paper look overambitious.<br>Consider if the aims can be simplified to focus the reader on key aspects of this study.	Thank you for this feedback! We have changed the section as follows:<br>*"In this study, we aimed to provide an in-depth analysis of changes to primary outcomes using a comprehensive nationwide sample over 9 years that represents the current practice of trial registration in academic research. Specifically, we aimed to…"*

79	METHODS	L152, 158. Why would a study be regarded as 'completed' if the trial has an 'Unknown status'? How can investigators be sure it was actually completed?	If a trial's registry entry is not updated for a certain amount of time, it is automatically set to 'Unknown' on ClinicalTrials.gov. However, the trials included in our sample were both past their primary completion date and had a results publication.
80	METHODS	L185. Please explain clearly 'If the reviewers could not find the correct publication, they excluded the publication'	We changed the text to explain this better:<br>*"For feasibility, we drew a random sample of 300 trials with accompanying results publications, from which three reviewers (MRH, MH, SY) extracted primary outcomes. Eight articles were excluded from the sample because they were not the main results publications (i.e., the final sample size was 292)."*
81	METHODS	L205. 'proportions': should be percentages?	We rephrased the text accordingly.
82	METHODS	L209. Why are these variables regarded as 'predictors'? Are future events being predicted from preceeding knowledge on study phase, sponsor, ... intervention?	We took the perspective of a reviewer or user of the trial publication who has the question "Which factor indicates that in this study an outcome change may exist?". However, to avoid confusion, we now use more neutral language and exchanged the terms with 'output variable' and 'input variables'.
83	RESULTS	L230. Consider expanding on 'changed to or from a secondary outcome'	We changed the wording to "*primary outcomes were added, dropped, changed to secondary outcomes, or secondary outcomes were turned into primary outcomes*".
84	RESULTS	L230. Percentages reported as integer values in Results text, but to 2 dec place in Tables. Please make consistent: report percentages in Tables as integers. It is unlikely that precision to 2 dec place of a percent is meaningful.	We changed all percentages with decimal places to integers, including supplementary tables and figure captions.
85	RESULTS	L259. I may have missed it, but the positive result for trials registered on ClincialTrials.gov seems an unusual finding and does not seem to be sufficiently explained. Is this finding because most trials tend to be registered on this registry anyway?	We added more context to the Discussion section about this and state:<br>*"The observation that the frequency of outcome changes declined over time may be explained by recent discussion on clinical trial reporting practices. Regarding the type of registry, the association might be explained by better usability of the ClinicalTrials.gov platform, which might facilitate more frequent changes to registry entries.*

			Regarding sponsorship, our results could indicate better reporting and higher transparency in industry-sponsored trials (which change their registry entries more often than non-industry-sponsored trials). We currently have no sufficient data to quantify the role of selective reporting bias in this sample."
86	DISCUSSION	Show paragaraph separation throughout, in the Discussion.	We increased the spacing between paragraphs.
87	DISCUSSION	L272. should be '[have] discrepancies'	We rephrased the text accordingly.
88	DISCUSSION	L288. Replace 'likelihood' with 'odds'. 'Likelihood' has a special meaning in statistics.	We rephrased the text accordingly.
89	DISCUSSION	L298. Reviewers already review on volunteer basis under time-poor conditions. What incentives are there for reviewers to do more work, as suggested here?	This is an important point, thank you. We certainly do not want to put more burden on peer reviewers. We changed the sentence to make it clear that we believe this should rather be done by specialized, paid personnel, not by volunteers:<br>*"Journals could implement editorial policies that require specialized personnel to assess the historical versions of submitted clinical trials."*
90	DISCUSSION	L310. 'defining added or omitted specificity': rephrase to make clear	We rephrased the text accordingly: "*We used a relatively broad definition of primary outcome changes, defining changes to the specificity of primary outcomes as 'minor' changes.*"
91	DISCUSSION	L323. Delete 'that'	We deleted it accordingly.
92	DISCUSSION	L325. Rephrase 'did not only reveal these research findings'; clunky.	Thank you! We deleted that part and rephrased the text accordingly: "*Our analysis also demonstrated the feasibility of an efficient workflow…*"
93	REFERENCES	Please check reference formatting is consistent -- there are some inconsistencies throughout<br>* Make all titles sentence case (e.g. ref 3)<br>* Remove month<br>* Check all abbreviated journal names are correct (e.g. ref 26)<br>* Fix formatting ref 37	We have now carefully revised the references and thank the reviewer for highlighting these issues.

94	TABLES	Table 1.<br>* 'Multiple categories' of what? What 'timepoints'?<br>* State 'outcome' if it refers to an outcome, not just 'primary' or 'secondary'<br>* 'aggregation' of what?<br>* 'significant parts' of what?<br>* What is the meaning of the curly brackets?<br>* Define RECIST	Thank you for the feedback. We have made numerous changes to the table to make it more understandable, addressing all these issues.
95	TABLES	Table 2<br>* Make separate headers for median (IQR) and N (%). Break the table afdter first 3 rows to add the N (%) header<br>* decrease width of 2nd column so row items in 1st column do not break across lines<br>* State 'Other or not provided' as 'Other or Not provided' to be consistent<br>* Drop the last row. The total score is the sum of percentages within rows, not percentages across all rows.	We changed the table accordingly.
96	TABLES	Table 3 and Supplementary Table 4.<br>* Report percentages as integers (i.e. no dec pla) to be consistent with manuscript<br>* Make formatting consistent: capitalise 'Recruitment', 'Post-completion', 'Post-publication'<br>* Define why font is bold<br>* Define statistics in last column	We changed the table accordingly.
97	TABLES	Supplementary Tables 1, 2<br>* Define statistics<br>* Report percentages as integers<br>* Make column labels consistent with capitalisation<br>* Why does 'p-Value' have a capital V?<br>* Define 'Intercept'<br>* Report p values less than 0.001 as 'p<0.001'; there's no need to show p values to 16 decimal places<br><br>Please pay more attention-to-detail in Tables and Figures.	Thank you. We made the requested changes to all the supplementary tables. To avoid confusion we dropped the term “intercept”.

98	FIGURES	Overall, I found the Figures the least clear and the hardest section of this manuscript to follow, which is a shame since so much information is conveyed in figures. Please address the comments.	Thank you for your very detailed and highly useful feedback!
99	FIGURES	Fig 1.<br>* Capitalise t in 'time'. Make 'Publication' single line<br>* In Legend, define vertical dotted lines, vertical dashed line, boundaries of blue box. Explain meaning of bidirectional red arows; why do arrows need to be red? Why does 'Journal publication' need an icon, but the other text does not?<br>* Expand the legend substantially to talk readers through the figure and orientate them. Each figure needs to 'stand-alone'.<br><br>Note, any qualitative difference in patterns in figures can be interpreted to have a meaning, otherwise no difference is needed. So if differences are shown but there is no meaning, it leaves the impression that the figure is sloppy and rushed, not clear and informative.	We carefully followed the reviewer's advice and now give more explanation, aiming to make the figures stand-alone and self-explanatory.<br>We also included an example (suggested by another reviewer) and updated our figure legend to reflect the suggested changes.
100	FIGURES	Fig 2.<br>* Why are lower 3 boxes in grey but the others in black? Make consistent<br>* In Exluded trials:<br>* rephrase 'publication' with 'published'<br>* why were time limits of 2 and 5 years chosen? Was this explained in the text?<br>* by definition, a clinical trial that is not randomised or does not have an intervention can't be a 'trial'. Does this suggest that observational studies are registered on ClinicalTrials.org? Would it be more accurate to use 'study' here?<br>* 'samples' should be 'registries'?<br>* Also, expand the Legend to provide details on information in boxes.	Thank you!<br>* We have now made all boxes black and have – in accordance with another reviewer's comments – changed the flowchart to one single stream.<br>* We rephrased the text<br>* We have also clarified the text parts regarding our exclusion criteria.<br>* We agree that our wording was misleading. Yes, non-interventional and non-randomized studies seem to be registered both on ClinicalTrials.gov and DRKS. We made that clear and changed our wording to be more precise.<br>* 'samples': We meant trials that were part of both research projects underlying the dataset we used and thus duplicated in the combined dataset. We changed the wording to reflect this. We also expanded the legend, as requested.
101	FIGURES	Fig 3. Very hard to follow. At present, the figure is so unintuitive and uninformative that it seems better to drop it than keep it in.<br>* Does the height of the bar have meaning? Are bars drawn to scale?	Thank you for your feedback! While we generally would be open to dropping the figure, we believe that it still conveys a lot of information, Thus, we moved it to the

		* Label the y axis, provide axis and axis markers.<br>* Join the bars so they are continuous<br>* Define 'Major' and 'Minor'. Define 'Start' vs 'Completion' I.e. start of what?<br>* Define 'Pub'<br>In legend,<br>* Explain, what is this figure meant to show? What are the bars supposed to represent, and what are the curved grey regions defined as and meant to show.<br>* Expand the legend substantially to explain this figure to readers.	supplement (now: Supplementary Figure S1) and improved the figure according to your suggestions:<br>We have joined the bars and added a scale so that the trials add up to 100%. We have also included a better description in the legend. Additionally, we have created an interactive online version of the plot, which we believe is easier to use and understand:<br>https://martin-r-h.shinyapps.io/invisibleoutcomechangesfigure/
102	FIGURES	Fig 4, and similar Supplementary Figs.<br>* This seems to be a specialised plot for a niche area, but will be new to general readership of this journal.<br>Add an introduction to the legned to explain how this figure is to be read and interpreted, what the vertical bar heights indicate, what the horizontal bar heights indicate, and what the dots and connecting lines indicate. Provide an example reading of the matrix.<br>* At the moment, I cannot match the numbers reported in the legend to bar heights etc. in the figure: e.g. where did 41 (14%) come from?	Thank you! We now completely revised these Figures, which we believe makes them easier to understand. We have also added a more thorough caption of these Figures and provide the reasoning for our number of 41 trials with invisible changes.
**NR**	**AREA**	**COMMENT (Reviewer 3)**	**RESPONSE**
	CRITICAL		
	General	I found this difficult to follow, I'm afraid. On this reading, I was left suspecting that readers will think: "Is this a German problem?", "Is this an old problem?" and/or "Is this a real problem?" I didn't fully understand what had been done nor, often, why. The comments are all intended constructively and I hope they come across that way. Below are listed detailed comments.	Thank you for your very clear and constructive feedback! We have addressed all the comments to improve the context and clarity of the project. We have specifically highlighted that the problem is new (without claiming primacy, as suggested by the Editor), underlined the relevance, and explain more detailed why our sample is from Germany and our reasons to believe the result can be generalized beyond this geographic reason and time period.
103	General	I'm afraid I didn't get the importance of this work. What am I missing? Is the suggestion that people don't change outcome measures if they	We have rephrased both our Abstract and our Introduction section to make the importance clearer.

		need to or that they report better or that registers or journals should take some action?	
104	General	I thought the terminology felt harsh and misleading, particularly "discrepancy" and "hidden". It's only discrepant if the paper differs to the most recent registry entry. It's only hidden if the change doesn't appear in the registry. (I spend most of my career researching how to improve the way do clinical trials - there's lots of scope for improvement - so I was surprised by how the wording of this manuscript provoked a defensive reaction in me.) This requires a big re-think, including in Table 1.	We appreciate your very helpful feedback! In line with comments by other reviewers, we exchanged the word 'hidden' with 'invisible', and hope it comes across as less accusing (which was not our intention). Please note that 'discrepancy' is a common term for changes to primary outcomes, which is also used in other publications on the topic. Still, we now consistently use the word 'change'. We also changed Table 1 to make it more understandable.
	MODERATE		
105	Background	Why is it a problem to look through the history of changes? This is the point of history of changes?	The problem is that the history of changes is not easily accessible and readable.<br>We now have further highlighted this in our Discussion and state: "*At the same time, in their current form, historical versions of registry entries are not easily accessible and readable, which makes checking them tedious.*"
106	Throughout	I always understand that an "outcome" is how the patient does and an "outcome measure" is how you measure the outcome. An endpoint would be the same as an outcome measure. The use of outcome (as a shorthand for "outcome measure"?) here seemed confusing.	We fully understand. However, the shorter version (e.g., 'outcome switching' instead of 'outcome measure switching') is quite established in related literature and discussions on the topic (please see e.g. the quotes from CONSORT that we report in the Introduction), and in our view it facilitates readability.<br>However, to be clearer, we consider this comment and now have explained this at the beginning of the manuscript: *"outcome measures (or, 'outcomes', for short)"*
107	Methods	:: Text ref :: "We based our analyses on two published datasets (from the IntoValue projects, 31,32) containing all interventional studies completed at German University Medical Centres between 2009 and 2017."<br>:: Comment :: Chosen for convenience rather than relevance?	Thank you for pointing us to this possible source of misunderstandings. We now better describe why we chose this sample, see comment #1 from the editor.

108	Methods	:: Text ref :: "The trials had been registered in either ClinicalTrials.gov or the Deutsches Register Klinischer Studien (DRKS), which is the WHO primary trial registry for Germany."<br>:: Comment :: How many were registered in both? I suspect quite a few. I would have liked the results broken down by registry: some have better supported updating the entries than others.	In our dataset, 11 out of 1402 trials registered on ClinicalTrials.gov have a cross-registration in the DRKS (0.78%), and 4 out of 344 trials registered in the DRKS have a cross-registration on ClinicalTrials.gov (1.16%).
109	Methods	:: Text ref :: " We included any study that: 1) has a registry entry in either the ClinicalTrials.gov or the DRKS database, 2) was completed between 2009 and 2017 according to the trial status described as 'Completed', 'Unknown status', 'Terminated', or 'Suspended' (ClinicalTrials.gov), or 'Recruiting complete, follow-up complete', 'Recruiting stopped after recruiting started', or 'Recruiting suspended on temporary hold' (DRKS), 3) reported in the registry that a German University Medical Centre was involved (i.e., mentioned as responsible party, lead/primary sponsor, principal investigator, study chair, study director, facility, collaborator, or recruitment location; for definitions see 31,32), 4) has published results, i.e., a full-text publication of the trial results was found by the search methods described in the IntoValue protocols (31,32), 5) is registered as a randomised trial."<br>:: Comment :: First, are these joined together with "and" statements or "or" statements? I suspect the form but it's not clear. Second, the selection criteria confuse "completing accrual" with "completing the trial". Which is actually meant?	Thank you for pointing this out! We mean "and" – we clarify this in the revision. We define trial completion according to the trial status (e.g., 'Completed', 'Unknown status', 'Terminated', or 'Suspended' in ClinicalTrials.gov).
110	Methods	:: Text ref :: "For feasibility, we drew a random sample of 300 publications, from which three reviewers (MRH, MH, SY) extracted primary outcomes."<br>:: Comment :: I accept couldn't do all of them but why 300?	Based on our experiences in the previous rating of within-registry changes, 200 trials per reviewer (due to duplicate assessment) was the maximal feasible amount. This sample still represents 17% of the total trials assessed.
111	Methods	:: Text ref :: "If the reviewers could not find the correct publication, they excluded the publication…"<br>:: Comment :: If it's not the right publication, how could discrepancy be judged?	Our work was based on a highly granular and large-scale dataset on both registry entries and accompanying results publications, which had been created in a previous project of our group. The results publications were identified by extensive manual searches. Still, there were some publications in the dataset which had been misclassified as results publications. In those very few cases, if no other results publication could be found by

			searching the trial title, the trials (n=8) were excluded. We modified the text to make this clearer (see also comment #80).
112	Results	:: Text ref :: "Of 1746 trials, 393 (23%) had an outcome discrepancy reported within the registry (Table 3; Figure 3), 142 trials (8%) had major discrepancies, i.e., a primary outcome was newly added, dropped, or changed to or from a secondary outcome."<br>:: Comment :: When? This is really important for the documented-history vs unreported-discepancy.	For this summary statistic we counted every change after first patient inclusion up to the latest entry. Additional information on changes between the key timepoints are visualized in Supplementary Figure S1 (formerly: Figure 3) and Figure 3 (formerly: Figure 4) and are described in the modified main text:<br>*"Of 1746 trials, 393 (23%) had a primary outcome change between trial start and latest registry entry version, i.e., within registry entry versions between key trial milestones (Table 3; Figure S1). Minor changes were found in 318 (18%) trials. 142 trials (8%) had major changes, i.e., primary outcomes were added, dropped, changed to secondary outcomes, or secondary outcomes were turned into primary outcomes. Of these major changes, 66 (4%) happened between first patient inclusion (start date) and trial completion date, 49 (3%) between completion and publication date, and 36 (2%) between the publication date and the latest registry entry."*
113	Results	Why look only at the results paper? Many trials will publish a methods paper too. The choices may be better documented in these papers (where there is space) than in results papers (where journals, particularly the well-read journals, are very restrictive in space).	We chose to assess results publications because current reporting guidelines such as CONSORT clearly state that deviations from the protocol should be reported in the results publication. Since outcome changes are among the most critical protocol deviations in trials, we agree with the CONSORT view. However, we also agree that methods papers or protocol papers may include such information, and have now added this in the Discussion and state:<br>*"Second, we only assessed reporting of outcome changes in the main results publications of the respective trials. These changes could also have been published in a protocol or methods paper. We assessed the results*

			publications as they have higher impact, and because current reporting guidelines such as CONSORT state that deviations from the protocol should be reported in the results publication."
114	Results	:: Text ref :: "Out of 292 randomly selected trials among the 1746 trials, 120 (41%; 95% CI [35%, 47%]) had a discrepancy between the latest registry entry and the publication, of which 54 (18%; 95% CI [14%, 237 23%]) were major and 75 (26%; 95% CI [21%, 31%]) were minor discrepancies."<br>:: Comment :: The 120 / 292 is the interesting part of this manuscript. This should be the focus. The rest reads a bit like noise to me. Broken by registry would be good.	Thank you for highlighting this issue. We agree that the prevalence of outcome changes between latest registry entry and publication is an important finding. However, this problem is known while we believe that more awareness of the prevalence of 'hidden' or 'invisible' changes is the finding that deserves the most focus in our manuscript.<br>Results broken down by registry are in Supplementary Tables 1 and 2 but would not change our interpretation of our main findings.
115	Discussion	:: Text ref :: "The ICMJE policy states that any changes to the registration should be explained by authors in the publication (4)."<br>:: Comment :: Why "the publication" rather than "as part of the publications"? ICJME aren't always right, of course. They don't enforce CONSORT, they are too strict on authors and they got data sharing wrong, so I suspect "a" rather than "the" should be fine.	Thank you for highlighting this. We made that change.
116	Discussion	:: Text ref :: "Still, some trials change their outcomes at two or even three different timepoints over the course of the trial."<br>:: Comment :: A reminder of examples here would be good. I've been involved recently with some very long-term trials. During the course of the trials, external projects showed that earlier outcome measures could be used as a surrogate for the existing primary outcome measures so the trial team (without reference to accumulating comparative data, after considering the implications for sample size and timelines and after peer review) formally amended the outcome measures. This saved a couple of years and helped patients sooner. The rational for the changes was documented in methodology papers and these were referenced in the main results papers. These trials were registered both in clinicaltrials.gov which allows changes to be captured and ISRCTN.com which doesn't allow updates (or didn't at the time). How would these examples be judged here.	To make the practice of changing outcomes at multiple timepoints more understandable, we updated Figure 1 to include an example. Thank you for pointing this out!<br>We would like to point out that we absolutely do not argue against outcome changes per se (we agree that they may be well necessary and encountered such situations in our own trials as well). We only highlight the problem of ***undisclosed*** outcome changes. We fully acknowledge this aspect and now added a short paragraph to the conclusion section:<br>*"To conclude, primary outcome changes are very common in clinical research. Such changes are often, in one of four cases, 'invisible' in the trial registries and almost never reported in publications. While changes to*

			primary outcomes are not unusual in clinical trials and may be well justified and sometimes even desirable, undisclosed changes are a problem."<br>Regarding methods papers, please see comment #113.
117	Discussion	:: Text ref :: "Interestingly, we found not only cases in which primary outcomes gained more detail, but also cases in which primary outcomes were described with less detail than before."<br>:: Comment :: I don't really know what this means.	We clarified our Table 2 (formerly: Table 1), which provides the terminology of outcome changes that we use to clarify this issue.
118	Figure 1	I didn't really understand this. Why does the blue box extend beyond the dotted line?	We have reworked Figure 1 to be clearer (see also comment #99 of Reviewer 2) and also added a better description in the caption.
119	Figure 2	This is not well presented. Sometimes a splitting line means the patients go down just one of the paths and other times it means the patients go down both.	To make our flowchart more understandable, we included everything in one single flow and added some descriptions, hoping that they make the flowchart more understandable.
120	Figure 3	No scale, no definitions, no timelines. I completely don't understand this.	We extensively revised the figure (see also comment #49 from the Editor and comment #101 from Reviewer 2).
121	Table 2	I would have liked a column for all trials (as shown) and an extra one for the 300 samples and another for the ones selected as odd.	We added an extra column to the table, containing the numbers for the random sample.
122	Table 3	What is a post-publication discrepancy? What differs from what in those instances? Is this a change in the registry after a first results paper in anticipation of another longer-term paper?	Thank you for this remark. We extended our explanation in the table captions.
123	Supp Tables	The lack of totals make these difficult to use. What are these p-values? And they are the separate by row rather than looking for patters without predictors?	We have revised our tables, including some added detail on what the p-values mean.
