# Supplementary material for: Frequency of multiple changes to prespecified primary outcomes of clinical trials completed between 2009 and 2017 in German university medical centers: A meta-research study
Source: PLoS Med. 2023 Oct 31;20(10):e1004306. doi: 10.1371/journal.pmed.1004306 (PMC10645365; doi:10.1371/journal.pmed.1004306)
Supplement: Attachment — Submitted filename: ASCERTAIN_Response_to_Reviewers_Changes_final.pdf [file pmed.1004306.s011.pdf]

Point-by-point reply
28 September 2023

NR	AREA	COMMENT (Editor)	RESPONSE
1	GENERAL	Thank you for your detailed responses to previous editor and reviewer comments. Please see below for further comments that we require you address in full prior to publication.	Thank you!
2	GENERAL	* We require that you refrain from any reference to 'hidden' or 'invisible' changes which could mislead the reader and implies an intentional lack of transparency. Indeed, you were able to find these changes in available documents. We suggest 'late changes' or 'subsequent amendments' or similar, as an alternative. In addition, the tone throughout is rather accusatory – there are many reasons why amendments may be necessary to trial protocols – a more objective and balanced presentation of only the facts supported by the data you present are required. This is a prerequisite to publication. *	Thank you for your feedback on this matter! We have now gone through the manuscript and removed all references to 'hidden' or 'invisible' changes. We instead opted for a more descriptive language, and we hope that the point is now sufficiently addressed.
3	GENERAL	** In quite a few studies, primary outcomes were modified AFTER recruitment began and, very few papers transparently reported protocol changes per ICMJE. Please clarify whether you checked when primary (or other) outcome changes occurred relative to data lock. This is a key point of the manuscript which we agree warrants specific discussion. Please include. **	Thank you, this is an important point. We have now clarified in the limitations section that we have no information about data lock:<br>*"Third, while we have information about changes that occurred post-completion, we have no data regarding changes in relation to the clinical trial's data lock, that is, the timepoint at which no further information is added to the dataset (which might lie after the completion date)."*
4	TITLE	Please revise your title to refrain from using the word 'invisible', we suggest, 'Frequency of changes to prespecified primary outcomes of clinical trials completed between 2009 and 2017 in German university medical centers: A meta-research study'.	As suggested, we have removed the term 'invisible' from the title. However, we feel that our focus on registry histories is a key point of our manuscript, which sets it apart from previous work in the area, and which would not be well represented by the title 'Frequency of changes…'<br>We thus changed the title to 'Frequency of multiple changes…', which we hope is sufficiently descriptive.<br><br>If the editorial team feels that our suggested title still does not work, we are happy to change it along your suggestion.
5	COMPETING INTERESTS	All authors must declare their relevant competing interests per the PLOS policy, which can be seen here: https://journals.plos.org/plosmedicine/s/competing-interests	After double-checking the PLOS policy, we concluded there are no financial or non-financial relationships that interfere with, or could reasonably be perceived as interfering with, the full and

		For authors with ties to industry, please indicate whether any of the interests has a financial stake in the results of the current study.	objective presentation or publication of our research. We thus have no competing interests to declare. We hope we sufficiently indicated this in the editorial manager but have also added a specific statement to the manuscript (which could be deleted in copyediting, of course).
6	ABSTRACT	Line 69 – in reference to 'major' changes – please briefly explain what is considered to be 'major' in this study.	We have explained the meaning of major changes in the first paragraph of the Abstract's Methods and Findings section. We have now repeated the explanation in the relevant section, which now reads as: *"Of all 1746 trials, 23% (n=393) had a primary outcome change between trial start and latest registry entry version, with 8% (n=142) being major changes, that is, primary outcomes were added, dropped, changed to secondary outcomes, or secondary outcomes were turned into primary outcomes."*
7	ABSTRACT	Line 76/77 – 'often major' could be considered an overstatement in light of the data presented. Please interpret the study based on the results presented in the abstract, emphasizing what is new without overstating your conclusions.	We changed this section as follows: *"In this study, we observed that changes to primary outcomes occur in 55% of trials, with 23% trials having major changes. They are rarely transparently reported in the results publication and often not visible in the latest registry entry version."*
8	AUTHOR SUMMARY	Line 87 – suggest 'allow assessment of…'	We have changed this accordingly in the Author Summary and also in the Abstract, where a similar expression was used.
9	AUTHOR SUMMARY	Line 90 – suggest 'unapparent' instead of invisible.	We changed the term accordingly, thank you for the suggestion! See also comment #2.
10	AUTHOR SUMMARY	Line 91-92 – suggest 'Our objective was to investigate how often primary outcomes are changed in the trial registry over the course of a trial and, what proportion of the changes were unapparent.'	We have revised the section as follows: *"Our objective was to investigate how often primary outcomes are changed in the trial registry over the course of a trial, and how often outcome changes are unapparent if one compares only the latest registry entry version to the publication."*
11	AUTHOR SUMMARY	Line 100 – please revise the use of the terms 'visible' and 'invisible'.	We have revised the section as follows: *"We observed changes that can be easily identified by comparing the results publication to the latest*

			registry entry in 41% of trials. In 14% of trials, however, the changes would require an in-depth look within the historical versions of that trial's registry entry."
12	AUTHOR SUMMARY	Line 102 – suggest 'Only 1% of changes (2 trials), were reported in the corresponding publications.	For more precision, we changed the sentence to:<br>*"Only 1% of trials with changes (2 trials) reported this in the corresponding publications."*
13	AUTHOR SUMMARY	Line 106 – as above how do you define 'major'? It would be helpful to include (brief) details under the 'what did the researchers do and find' section.	We have added a new bullet point with a definition:<br>*"We defined adding or dropping primary outcomes, changing them to secondary outcomes, or turning secondary outcomes into primary outcomes, as major changes."*<br><br>At the same time, we merged the first two bullet points so that number of bullet points does not become too large. For consistency, we also added the number of 'major' changes to the Author Summary, which we had not reported before:<br>*"We found that approximately 55% of trials had primary outcome changes at any timepoint over the course of a trial; 23% of trials had major changes."*
14	AUTHOR SUMMARY	Line 107 – please remove the word 'invisible'.	We have revised the section as follows:<br>*"More transparency is needed, supported by deeper analysis of registry entries to reveal these outcome changes."*
15	INTRODUCTION	Throughout, please remove reference to 'invisible'.	We have carefully gone through our manuscript and removed all references to 'invisible' changes, instead using a more descriptive language. See also comment #2.
16	INTRODUCTION	Line 113 – suggest 'validity' or similar, in place of 'trustworthiness'.	We changed it accordingly.
17	METHODS and RESULTS	Throughout please remove all reference to 'invisible'.	We have carefully gone through our manuscript and removed all references to 'invisible' changes, instead using a more descriptive language. See also comment #2.
18	METHODS and RESULTS	Line 202 onwards – you only briefly refer to the classification system you use to determine 'severity' of the change identified but, you give no details other than referring the reader to the table. It is a little unfair to expect the reader to look elsewhere for this. Please provide brief details in the text.	We have changed the text accordingly:<br>*"Reviewers assessed changes to the primary outcomes between the four key trial milestones within the registry (Fig 1), using a classification system that categorizes changes to primary outcome as either major changes (based on an existing*

27			*classification system [40]) or minor changes (adapted from [41] and [42]). Major changes were defined as adding or dropping primary outcomes, changing them to secondary outcomes, or turning secondary outcomes into primary outcomes. Minor changes were defined as changes to the outcome's type of measurement, metric or method of aggregation, or timing of assessment, or as either added specificity or omissions to these (for more detail see Table 2)."*
19	DISCUSSION	Throughout please remove all reference to the term 'invisible'.	We have carefully gone through our manuscript and removed all references to 'invisible' changes, instead using a more descriptive language. See also comment #2.
20	DISCUSSION	The editorial team agree that you should present a more balanced and nuanced discussion of the drivers of protocol/primary outcome changes, the very vast majority of which are legitimate.	We have revised our text to be more nuanced and more balanced.
21	DISCUSSION	It would also be helpful to contextualize the discussion in context of the 'severity' scoring system you apply to the changes that you identify.	Thank you, we added the following to our text:<br>*"We used a relatively broad definition of primary outcome changes, defining changes to the specificity of primary outcomes as 'minor' changes. Our broader definition and inclusion of more 'minor' outcome changes, some of which might not constitute a large threat to a trial's validity, might have led to our higher estimates."*
22	DISCUSSION	Line 370 – would 'not uncommon' be a better term?	We changed it accordingly.
23	DISCUSSION	Line 371 – 'often' is somewhat subjective and perhaps an overstatement. Suggest simply stating 'one in four'.	We changed it accordingly.
24	REFERENCES	Please ensure that all web references include an 'Accessed [date]' instead of, 'Cited [date]'. Please check and amend throughout.	We changed this throughout our references.
25	REFERENCES	Please remove all instances of '[internet]'.	We changed this throughout our references.
26	SUPPORTING INFORMATION	Table S1 - please detail the meaning of the asterisk against the p values in an appropriate footnote.	Thank you, we added a footnote explaining what the asterisk means:<br>*"Asterisks indicate p-values below the common threshold for statistical significance, that is, p < 0.05."*
27	SUPPORTING INFORMATION	Table S4 – please revise 'No' to 'N' (or use the full word 'number') and define in the footnote.	To avoid any confusion with the denotation of our sample N, we opted for using the descriptive term "Number" throughout all tables. We also now consistently use "%" within all tables, instead

			of “percentage”, and “95% CI” instead of “confidence interval”.
28	SUPPORTING INFORMATION	PRISMA Checklist – thank you for including the checklist. Please revise the final column to detail section and paragraph numbers as opposed to page numbers, as these often change at publication.	Thank you! We have amended the PRISMA Checklist accordingly.
29	SOCIAL MEDIA	To help us extend the reach of your research, please detail any Twitter handles you wish to be included when we tweet this paper (including your own, your coauthors’, your institution, funder, or lab) in the manuscript submission form when you re-submit the manuscript.	Below, please find our Twitter (or X) handles, which we believe we also provided in the submission system:<br>@MartinHolst9<br>@m_haslberger<br>@Strech_Da<br>@LGHemkens<br>@questbih<br>@berlinnovation<br>@ChariteBerlin
**NR**	**AREA**	**COMMENT (Reviewer 1)**	**RESPONSE**
30	GENERAL	Thank you to the authors for addressing my previous comments well. I have no further issues to raise.	Thank you, we are happy to hear that we could sufficiently address your remarks.
**NR**	**AREA**	**COMMENT (Reviewer 2)**	**RESPONSE**
31	GENERAL	The authors have made substantial changes to the manuscript. A few smaller edits for clarity remain.	Thank you!
32	ABSTRACT	L46. Rephrase as: Clinical trial registries [edit: allow to assess -> make transparent] deviations…	We chose to follow the Editor’s suggestion more closely (see comment #8), rephrasing the sentence as:<br>*“Clinical trial registries allow assessment of deviations of published trials from their protocol”*
33	ABSTRACT	L56-60. Rephrase so sentence stem reads consistently with all enumeration points<br>i.e. We determined changes of primary outcomes … and (3) [edit: 'invisible' changes … -> that occurred after trial start with no change between last registry entry version and publication (i.e. invisible changes) …]	Thank you for noticing this! We rephrased the sentence, which now reads as follows:<br>*“We determined changes of primary outcomes (1) between different versions of registry entries at key trial milestones, (2) between the latest registry entry version and the results publication, and (3) changes that occurred after trial start with no change between latest registry entry version and publication (so that assessing the full history of changes is required for detection of changes).”*
34	AUTHOR SUMMARY	L86. Rephrase: They [edit: allow to assess -> make transparent] how closely…	We chose to follow the Editor’s suggestion more closely (see comment #8).
35	INTRODUCTION	L132-134. Rephrase sentence: [edit: Only a closer look … not easily accessible -> These changes would only	Thank you! We rephrased the sentence according to your suggestion:

		be discovered on closely investigating the histroy of changes in trial registries.]	*"These changes would only be discovered by closely investigating the history of changes in trial registries, which some trial registry platforms maintain. These histories, however, are often not easily accessible."*
36	REFERENCES	Please check all refs are formatted consistently.	We have gone through all references, checked them, and changed them where necessary.
37	REFERENCES	Numerous refs need the article title changed to sentence case e.g. Refs 3, 4, 6, 9, 11, 12, ...	We have gone through all references, checked them, and changed them where necessary. All references should be sentence case now.
